# An effect of large-scale deletions and duplications on transcript expression

**DOI:** 10.1007/s10142-022-00946-5

**Published:** 2022-12-23

**Authors:** Magda Mielczarek, Magdalena Frąszczak, Anna E. Zielak-Steciwko, Błażej Nowak, Bartłomiej Hofman, Jagoda Pierścińska, Wojciech Kruszyński, Joanna Szyda

**Affiliations:** Wroclaw University of Environmental and Life Sciences, Kozuchowska 7, 51-631 Wroclaw, Poland

**Keywords:** CNVs, DNA-seq, Transcript expression, RNA-seq, *Sus scrofa*

## Abstract

Since copy number variants (CNVs) have been recognized as an important source of genetic and transcriptomic variation, we aimed to characterize the impact of CNVs located within coding, intergenic, upstream, and downstream gene regions on the expression of transcripts. Regions in which deletions occurred most often were introns, while duplications in coding regions. The transcript expression was lower for deleted coding (*P* = 0.008) and intronic regions (*P* = 1.355 × 10^−10^), but it was not changed in the case of upstream and downstream gene regions (*P* = 0.085). Moreover, the expression was decreased if duplication occurred in the coding region (*P* = 8.318 × 10^−5^). Furthermore, a negative correlation (*r* =  − 0.27) between transcript length and its expression was observed. The correlation between the percent of deleted/duplicated transcript and transcript expression level was not significant for all concerned genomic regions in five out of six animals. The exceptions were deletions in coding regions (*P* = 0.004) and duplications in introns (*P* = 0.01) in one individual. CNVs in coding (deletions, duplications) and intronic (deletions) regions are important modulators of transcripts by reducing their expression level. We hypothesize that deletions imply severe consequences by interrupting genes. The negative correlation between the size of the transcript and its expression level found in this study is consistent with the hypothesis that selection favours shorter introns and a moderate number of exons in highly expressed genes. This may explain the transcript expression reduction by duplications. We did not find the correlation between the size of deletions/duplications and transcript expression level suggesting that expression is modulated by CNVs regardless of their size.

**Supplementary Information:**

The online version contains supplementary material available at 10.1007/s10142-022-00946-5.

## Introduction

Copy number variation (CNV) has been recognized as an important source of genetic variation (Gamazon et al. 2011; Jiang et al. 2014). Many studies were conducted in mammals, particularly in humans and rodents, to link structural variation to phenotypic variation and disease susceptibility (Stankiewicz and Lupski 2010; Yalcin et al. 2011). Potentially, CNVs exhibit a high impact on phenotypes, by changing gene structure and dosage, altering gene regulation, and exposing recessive alleles (Mills et al. 2011; Geistlinger et al. 2018). In pigs, it has been found that genes related to olfaction and neurological processes (Paudel et al. 2013, 2015), coat colour (Giuffra et al. 2002), diseases (e.g. umbilical hernia; (Long et al. 2016)), and production performance (Jiang et al. 2014; Xie et al. 2016) are enriched in CNVs. However, compared to humans and model organisms, relatively few studies of CNVs have been carried out in pigs. The Database of Genomic Variants (DGVa, www.ebi.ac.uk/dgva) comprises CNVs from only four studies: nstd24 identifying 37 variants (Fadista et al. 2008), nstd44 reporting 49 variants (Ramayo-Caldas et al. 2010), nstd138 with 223,216 variants (Li et al. 2017), and nstd117 with 737 variants (Long et al. 2016). During the last few years, CNVs in swine genomes were mostly detected using aCGH approach (Fadista et al. 2008; Wang et al. 2014) or SNP arrays (Ramayo-Caldas et al. 2010; Wang et al. 2012, 2013; Chen et al. 2018). Nevertheless, the development of next-generation sequencing (NGS) technology during the past decade has created new, more accurate tools of CNV detection at a base-pair resolution (Rubin et al. 2012; Paudel et al. 2013; Esteve-Codina et al. 2013; Liu et al. 2018).

Since CNVs play an important role in genomic studies, and pigs are one of the most economically important livestock species worldwide as well as a model organism for many human diseases (Meurens et al. 2012; Wang et al. 2014), the understanding of how CNVs act on transcript expression is essential. The availability of whole-genome and whole-transcriptome sequences allows for genome-wide identification of polymorphic variants and transcript expression levels. This project aims to characterize the impact of CNVs on the expression on transcript-level resolution.

## Results

### CNV calling pipeline and genomic annotation results

**Fig. 1:**
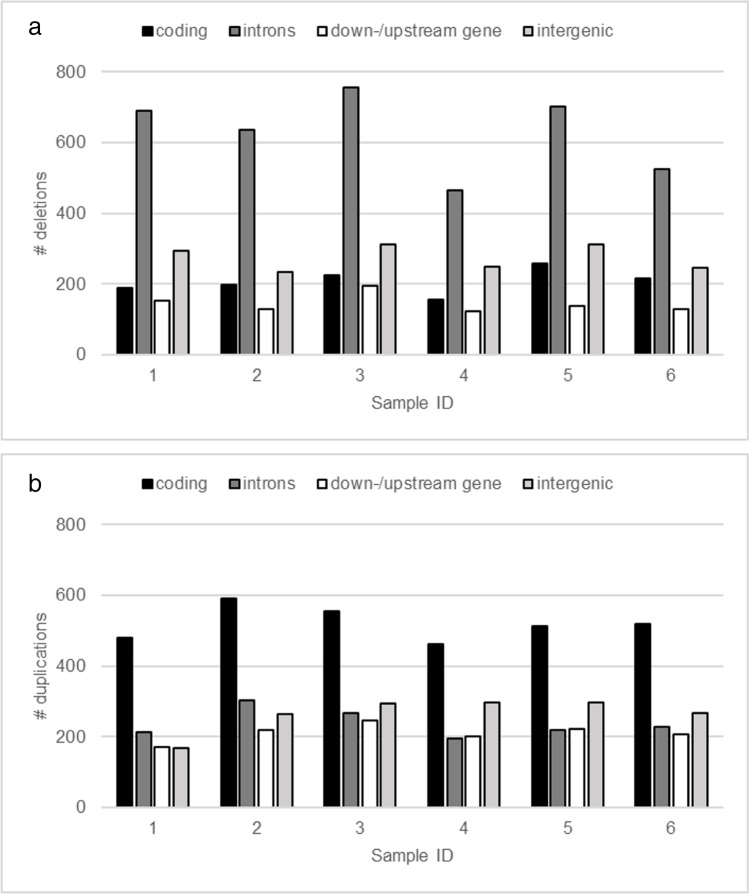
The number of **a** deleted and **b** duplicated genomic regions per individual

From 93,45 to 93,84 (DNA-seq) and from 87,15 to 91,45 (RNA-seq) percent of reads survived the cleaning procedure (details in the supplementary material S1, S3 and S4). The percentage of reads aligned to the reference genome for the six Polish Landrace boars was very similar and ranged between 98.16 and 98.49% with the percent of properly paired reads ranging between 93.99 and 95.15% (supplementary material S2). The average genome coverage after alignment varied from 17 to 21. The total number of deletions per individual ranged between 551 and 730, and duplications from 619 to 693. The length of deletions varied from 300 to 530,600 bp with the average of 3939 ± 10,976 bp and of duplications from 900 to 346,200 bp with the average duplication being 12,377 ± 20,154 bp long. Deletions occurred most often in introns (465–756 deletions per individual), followed by intergenic regions (235–312), coding regions (156–259), and up-/downstream genes regions (123–194) (Fig. 1a). Duplications were most common in coding regions (463–591) and introns (196–304) followed by intergenic (169–297) and upstream/downstream gene regions (173–246) (Fig. 1b).

### CNV impact on gene expression

**Fig. 2:**
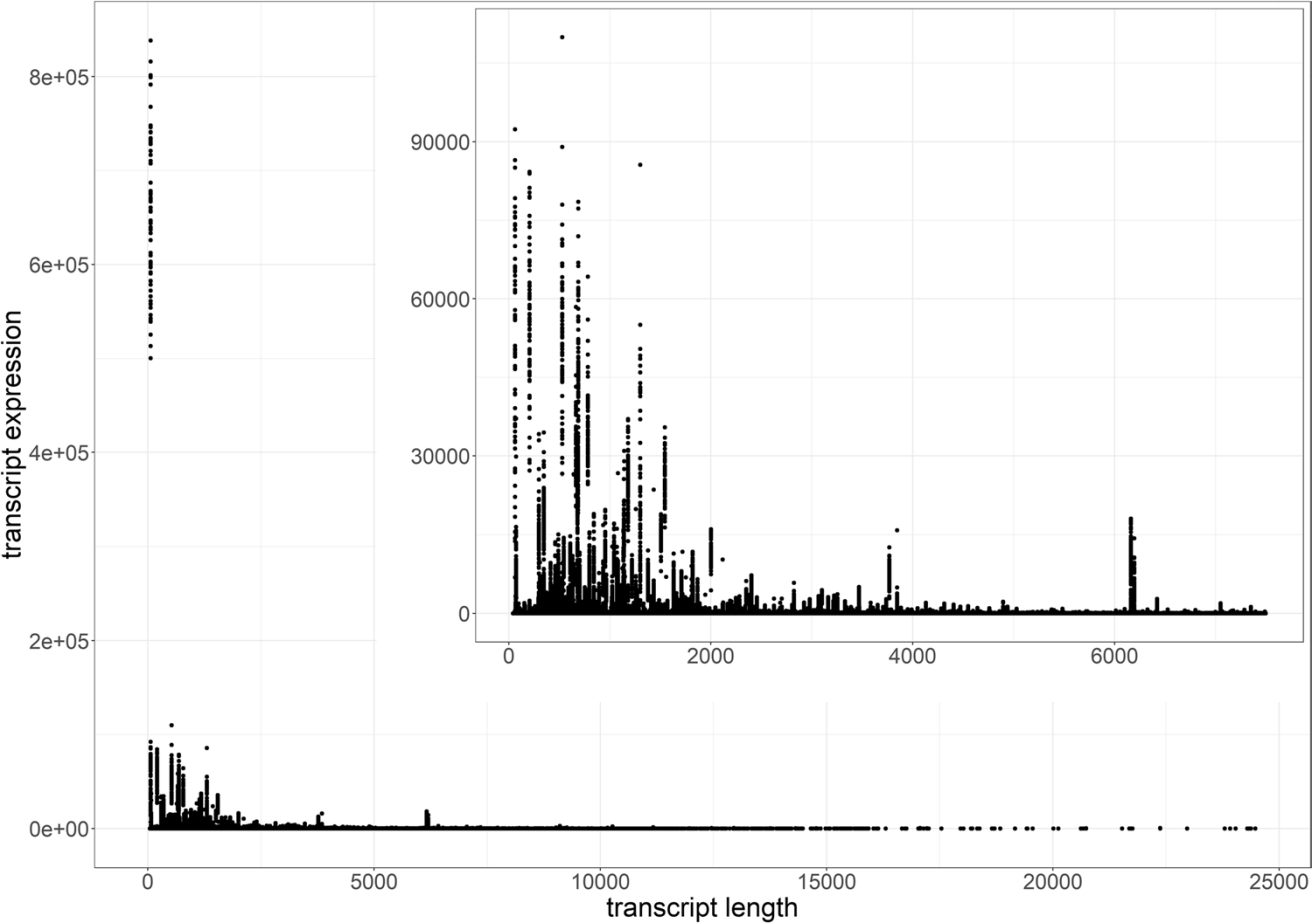
The expression levels and transcript lengths based on transcriptomic data of 143 sows

Random transcripts were selected to check if decreased/increased expression is not caused by the animal effect. The average expression of a random transcript that does not contain a CNV in 3 out of 6 animals was equal to the average expression of a random transcript without a CNV in the other 3 out of 6 animals (*P* = 0.456). While comparing mean transcript expression between individuals carrying CNV and those not carrying CNV in the particular transcript, we observed that transcript expression was significantly lower when a part of a coding region (*P* = 0.008) or an intron (*P* = 1.355 × 10^−10^) was deleted. Regions located upstream or downstream genes were not significantly altered by deletions (*P* = 0.085). For none region, the expression was increased by duplication (*P* = 0.990 for coding, *P* = 0.846 for intronic, and *P* = 0.872 for upstream and downstream gene regions). On the other hand, duplications of coding regions significantly decreased their expression levels (*P* = 8.318 × 10^−5^), while having no effect on expression when occurring in other regions. Since expression was decreased by duplication, we further explored the phenomenon of an effect of transcript length on its expression level based on longissimus dorsi muscle transcriptomic data of 143 sows (PRJNA403969) (Velez-Irizarry et al. 2019). There was a negative correlation between the length of the transcript and the transcript expression level (the correlation coefficient *r* =  − 0.27, *P* = 0). The level of expression decreases exponentially with increasing transcript length (Fig. 2).

**Fig. 3:**
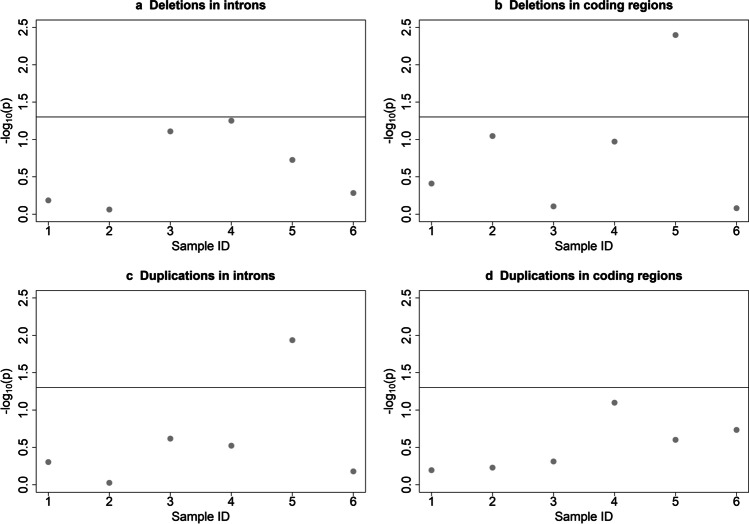
*P*-values referring to the correlation between the transcripts expression level and the size of **a** deleted fragments in introns, **b** deleted fragments in coding regions, **c** duplicated fragments in introns, and **d** duplicated fragments in coding regions. The horizontal line shows the cut-off for rejection of the null hypothesis (alpha = 0.05)

No consistent pattern was found regarding the correlation between the percent of deleted or duplicated transcript and transcript expression level (expressed as a difference between average expression in animals with CNV and animals with unaltered sequence). The correlation was not significant for all concerned genomic regions in five out of six animals except deletions in coding regions (*P* = 0.004) and duplications in introns (*P* = 0.01) for sample number 5. All *P*-values were presented in the Fig. 3.

## Discussion

### CNVs modulate transcript expression

CNVs contribute to the variation of gene expression in multiple ways including gene dosage effects, gene coding region disruption and deletion, or duplication of regulatory sequences (Stranger et al. 2007). It has been shown that CNVs alter not only gene expressions but also the timing of expression throughout the life of an organism (Chaignat et al. 2011). In humans, a positive correlation between CNV and gene expression in gastric cancer was found (Cheng et al. 2012). Moreover, it has been reported that CNVs induce gene expression changes in breast tumour tissues (Kumaran et al. 2017). Copy number loss and corresponding downregulation of gene expression was also found by Zhao and Zhao (2016) in human tumour suppressor genes. Moreover, considering particular genes, deletions decreased their expression resulting in disease phenotypes, e.g., retinitis pigmentosa (deletion of an intron of the PRPF31 gene) (Ruberto et al. 2021), DiGeorge syndrome (deletions within several genes) (Sellier et al. 2014), or Fanconi anaemia (deletion in exons of the FANCA gene) (Tischkowitz et al. 2004). Results confirming downregulation of gene expression by deletions are in line with observations made in present study on pigs. We hypothesize that deletions imply severe consequences by interrupting genes not only in coding but also in intronic regions. The latter may be explained by the fact that introns modify the expression level of their host gene in many different ways (Chorev and Carmel 2012) and for variety of genes important sequences that regulate expression are situated not in the promoter but rather are located within introns (Rose 2018).

Regarding duplications of coding regions, we observed a significant reduction of transcription expression. This is consistent with Qian et al. (2010) who reported that gene expression in yeast and mammals showed a substantial decrease after duplication. The authors explained that most of the reductions are neutral in the context of expression, but some are beneficial to rebalancing gene dosage after duplication. The dosage rebalancing hypothesis was also supported by Rogozin (2014), who stated that gene copy-number variations gains had a positive or negative dosage effect depending on the tissue type or environmental conditions. It means that balancing of positive and negative dosage effects is an essential factor causing diversification of expression patterns (rebalancing of expression) of duplicated genes in the course of fixation of gene duplications. Since expression was decreased by duplication, we also explored the effect of transcript length on its expression level. Our results demonstrate negative correlation between transcript length and its expression. High expression of short genes and lower expression of longer genes were reported in the literature. For example, to minimize the cost of transcription and other molecular processes such as splicing, natural selection may favour shorter introns in highly expressed genes (Castillo-Davis et al. 2002; Urrutia and Hurst 2003). Furthermore, highly expressed genes contain a moderate number of exons and produce shorter mRNAs with shorter 3′-UTRs (Chiaromonte et al. 2003). Moreover, Brown (2021) explained that transcription is a time-dependent process and it is expected that gene expression will be inversely related to gene length. This was confirmed by the author since high expression was found only among short, not among long genes, but at the same time, it was emphasized that the level of a gene’s expression can be affected by the tissue where the gene is transcribed. The complex analysis of genes length was also extensively investigated by Lopes et al. (2021). In the context of gene expression, the author stated that for shorter genes, their association with high levels of expression is not entirely correct and there is great variability of expression values among them. Instead, it was hypothesized that longer genes tend to be associated with functions important in the early development stages, while functions of shorter genes are important throughout the whole life (e.g. related to the immune system).

In the context of livestock, Park et al. (2014) did not observe a significant impact of copy number deletions on gene expression in race horses, but Lee et al. (2021) suggested that CNV can alter gene expression and affect multiple economically important traits in cattle. Moreover, Geistlinger et al. (2018) also pointed out the role of CNVs in the modulation of bovine gene expression by observing the effect of gene dosage in several immune genes and genes involved in major skeletal muscle pathways. The authors also concluded that CNV-associated expression may be manifested at the phenotypic level. In pigs, the ear size (one of the conformation traits that distinguish breeds) is modified by the 38.7-kb long CNV in the MSRB3 gene (Chen et al. 2018).

In relation to intergenic variants, little is known about their biological function in the genome (Antúnez-Ortiz et al. 2017). According to Chaignat et al. (2011), gene expression is a complex process depending on many factors including CNVs, and they were shown not only to modify the expression of genes that map within them but also located on their flanks and sometimes those at a great distance from their boundaries. They may even have effects on the gene whose distance can be as far as 1 Mb away by interacting with the functionality of the regulatory region like disrupting the interaction of the transcriptional unit with the promoter unit or by a change in the chromatin structure (Stranger et al. 2007; Klopocki and Mundlos 2011). For example, in the long-tailed macaque genome, a popular animal model in biomedical research, the 13-kb copy number loss of the intergenic region on chromosome 19 modulated the expression levels of neighbouring genes providing a selective advantage to environmental conditions. The results were significant for the kidney and the heart but not for the liver, lung, and spleen tissues. What is interesting, deletion affects expression of 45 genes that are encoded up to 1 Mb upstream and downstream of this CNV (Heckel et al. 2015). Although specific CNVs affecting expression upstream and downstream of these CNVs are known, it is worth to remember that intergenic CNVs are likely to better reflect neutrality than gene-containing CNVs (Perry 2008). This is in line with the results of our study where we investigated the overall contribution of intergenic CNVs in transcript expression modification which was not significantly changed by them.

## Conclusions

In the presented study, we investigated CNV’s impact on transcript expression and observed that deletion of coding and intronic regions reduce expression. We hypothesize that deletions imply severe consequences by interrupting genes. The significant decreasing impact of gene sequence duplication was also confirmed by the analysis of our data. Moreover, we estimated the negative correlation coefficient between the size of the transcript and its expression level. It is consistent with the hypothesis that selection favours shorter introns and a moderate number of exons in highly expressed genes. Nevertheless, we did not find a correlation between the size of deletions/duplications and transcript expression level suggesting expression is modulated by CNVs regardless of polymorphisms size.

## Materials and methods

### Material

The dataset comprised whole genome DNA and RNA samples of six Polish Landrace boars from *longissimus dorsi* muscle. For whole genome sequencing, 100 ng of DNA was used to prepare the library with a TruSeq DNA Nano (Illumina) Kit. The total number of 150-bp-long reads varied from 328,516,078 to 404,898,834. The average genome coverage ranged from 20 to 24 × per sample. In the case of transcriptomic data, libraries were produced from 50 ng of RNA fragments using KAPA RNA HyperPrep (HMR) (Roche) Library Preparation Kit. Sequencing yielded libraries with an average size of 297 million reads per sample (from 241,605,646 to 337,017,230 reads), and the read length was 100 bp. Both, DNA- and RNA-seq data were generated using Illumina NovaSeq 6000 in the paired-end module. The exact number of reads per sample can be found in the supplementary material S1. Genomic and transcriptomic data of six Polish Landrace boars are deposited in the NCBI Sequence Read Archive with BioProject Number PRJNA804745. Moreover, we used transcriptomic data of 143 females (PRJNA403969), generated using Illumina Hiseq 2500 from *longissimus dorsi* muscle (Velez-Irizarry et al. 2019). The whole swine transcriptome required to perform transcript expression quantification was taken from the Ensembl database release 104 (Howe et al. 2021). The animal research ethics committee approval was not required since meat samples were taken immediately after the slaughter in the slaughterhouse.

### CNV detection and expression quantification pipelines

Two bioinformatics pipelines were designed. (1) The *CNV detection pipeline* applied for DNA-seq data consisted of (i) quality control and data filtering, (ii) alignment of reads to the reference genome, (iii) quality control and data processing after alignment, (iv) CNV detection and filtration, and (v) CNV genomic annotation. The quality control was performed using the FastQC (bioinformatics.babraham.ac.uk/projects/fastqc) and MultiQC programs (Ewels et al. 2016). The Trimmomatic (Bolger et al. 2014) software was used to trim poor quality sequences by scanning each read with a 4-base sliding window and cutting them when the average quality per base dropped below 20 (the “SLIDINGWINDOW:4:20” option). The minimum length of read after trimming was set as 60 bp (“MINLEN:60”). The alignment to the Sscrofa11.1 (the assembly version: https://ftp.ensembl.org/pub/release-102/fasta/sus_scrofa/dna/) reference genome was performed by the BWA-MEM software (Li and Durbin 2009) with default parameters. Standard, post-alignment processes included sorting and indexing of reads; PCR duplicates removing and quality control with generating an alignment report. The average genome coverage was calculated using bedtools (Quinlan and Hall 2010). Two CNV detection tools, CNVnator (Abyzov et al. 2011) and Pindel (Ye et al. 2009), were used for variant identification. The algorithm implemented into the CNVnator software is based on the comparison of genome coverage (read-depth, RD) and assumes that regions with coverage different than the genome average correspond to CNVs (Medvedev et al. 2010). The size of the comparison-bin was set to 100 bp, which according to Abyzov et al. (2011), is recommended for samples with the approximate coverage of 20 × , as it was the case in our data. As a consequence, the CNVnator software provided a detection resolution of 200 bp of upstream and downstream CNV breakpoint positions. The split-read (SR) approach, implemented into the Pindel software, identifies CNVs by searching for read pairs in which one read from a pair is aligned to the reference genome at a unique position and its mate read is aligned only partially or is aligned to different regions of the genome. The raw outputs of both programs were edited by discarding variants outside the length range 50–1,000,000 bp. The CNV selection was performed by determining variants overlapping between both programs. The length-edited output of the CNVnator was used as a baseline dataset, which was validated by the length-edited output of the Pindel to identify overlapping regions. Each variant detected by the CNVnator, which was fully covered by Pindel (± 100 bp), was classified as validated. Moreover, to exclude false positive variants being a consequence of artefacts of the reference genome, CNVs overlapping with gaps in the reference genome were filtered out. Genomic annotation was carried out using the Variant Effect Predictor software (McLaren et al. 2016) to identify CNV located within genes or upstream and downstream gene regions up to 5000 bp. The upstream variant relates to the variant located upstream of the transcript start site (TSS) and the downstream variant to the downstream of the transcript termination site (TTS). If deletion/duplication overlapped more than one region (e.g., exon and intron) of the same transcript, it was not considered in this study.

(2) The *expression quantification pipeline* for transcriptomic data covered:

(i) quality control and data filtering; (ii) transcript quantification and expression normalization. Based on the FastQC report, we set the parameters for data filtering. The Trimmomatic software was used for filtering of low-quality data in the same way as described in the DNA-seq based pipeline above. Additionally, Illumina adapters were removed (“ILLUMINACLIP”). Transcript quantification and expression normalization were done by the Kallisto software (Bray et al. 2016) resulting in the expression expressed as TPM (transcripts per million).

### Statistical analysis

Statistical analysis was performed based on the validated CNV dataset, separately for duplications and deletions. The Kolmogorov–Lilliefors test was used to test the null hypothesis that expression data comes from a normally distributed population. The null hypothesis that expression of transcripts overlapping with CNV is equal to unaltered ones was tested using the Wilcoxon-signed rank test:

$$\mathrm W=\frac12\sum\limits_{\mathrm i=1}^{\mathrm n}\overline{\mathrm r}\left({\mathrm D}_{\mathrm i}\right)+\frac{\mathrm n(\mathrm n+1)}4$$

where $\overline{\mathrm r}\left({\mathrm D}_{\mathrm i}\right)$ denotes the rank of absolute values $\left|{\mathrm D}_{\mathrm i}\right|=\vert{\mathrm Y}_{\mathrm i}-{\mathrm X}_{\mathrm i}\vert$ with sign corresponding to the sign of the difference (${\mathrm Y}_{\mathrm i}-{\mathrm X}_{\mathrm i})$. The $\mathrm n$ is the number of transcripts overlapping deletions/duplications for at least one and not more than five (out of six) considered animals. ${\mathrm X}_{\mathrm i}$ denotes the mean TPM value for $\mathrm i$ th transcript for pigs with deletion/duplication identified as a part of this transcript, while ${\mathrm Y}_{\mathrm i}$ represented the average expression in individuals with the unaltered $\mathrm i$ th transcript. The tests were performed separately for coding, intronic, and upstream/downstream gene regions. Additionally, for each individual randomly located transcripts non-overlapping with CNVs were chosen. Since each transcript was represented by six TPM values (one per animal), we randomly divided these values into two groups and the mean expression for each group was calculated. To check if the lower/higher expression is not an effect of animal, the hypothesis that there is no difference in expression level between those two groups was tested. The whole procedure was repeated 500 times.

The Spearman rank correlation between the percentage of transcript covered by CNV and the transcript expression level was estimated and tested for being different than zero, what denotes independence:

$$\mathrm T={\mathrm R}_{\mathrm S}\sqrt{\frac{\mathrm n-2}{1-\mathrm R_{\mathrm S}^2},}$$

where,

$${\mathrm R}_{\mathrm S}=1-\frac{6\sum_{\mathrm i=1}^{\mathrm n}{({\mathrm R}_{\mathrm i}-{\mathrm S}_{\mathrm i})}^2}{\mathrm n(\mathrm n^2-1)},$$

${\mathrm R}_{\mathrm i}$ denotes the ranks of the percentage of transcript overlapping with deletion/duplication and ${\mathrm S}_{\mathrm i}$ denotes the ranks of the difference between mean TPM expression for a given transcript in a group with CNV overlapping transcript and in a group with an unaltered sequence. The null hypothesis of the test can be approximated by the *t*-Student distribution with $\left(\mathrm n-2\right)$ degrees of freedom. Additionally, the Spearman correlation coefficient between transcript length and the transcript expression level was estimated based on transcriptomic data of 143 individuals. The significance of the correlation coefficient was tested, using the same test as described above. The R package (R Core Team 2022) was used to perform computations and create graphics.

## Supplementary Information

Below is the link to the electronic supplementary material.

Supplementary file1 (PDF 278 KB)

## Data Availability

Genomic and transcriptomic data of six Polish Landrace boars are deposited in the NCBI Sequence Read Archive with BioProject Number PRJNA804745. Transcriptomic data of the 143 individuals can be found under PRJNA403969 identification number.
