# Supplementary material for: An effect of large-scale deletions and duplications on transcript expression
Source: Funct Integr Genomics. 2022 Dec 23;23(1):19. doi: 10.1007/s10142-022-00946-5 (PMC9789009; doi:10.1007/s10142-022-00946-5)

Article title: An effect of large-scale deletions and duplications on transcript expression. Journal name: Functional & Integrative Genomics. Authors: Magda Mielczarek[1], Magdalena Frąszczak[1], Anna E. Zielak-Steciwko[1], Błażej Nowak[1], Bartłomiej Hofman[1], Jagoda Pierścińska[1], Wojciech Kruszyński[1], Joanna Szyda[1].Affiliations: [1]Biostatistics group, Department of Genetics, Wroclaw University of Environmental and Life Sciences, Wroclaw, Poland. magda.mielczarek@upwr.edu.pl

Supplementary material S1. The number of DNA and RNA reads per sample before and after (filtered) the cleaning procedure.

**Sample ID**	**DNA raw reads number**	**Filtered DNA reads number**	**Filtered DNA reads (%)**	**RNA raw reads number**	**Filtered RNA reads number**	**Filtered RNA reads (%)**
1	388,601,958	364,654,182	93.84	241,605,646	213,643,886	88.43
2	354,408,466	331,996,222	93.68	256,334,754	223,403,442	87.15
3	404,898,834	378,398,058	93.45	311,677,634	285,032,944	91.45
4	350,988,196	329,260,198	93.81	308,285,870	280,382,394	90.95
5	356,551,276	334,140,458	93.71	329,032,224	293,273,448	89.13
6	328,516,078	306,895,952	93.42	337,017,230	304,770,528	90.43

Supplementary material S2. The percent of mapped and properly paired reads per sample.

**Sample ID**	**Mapped reads (%)**	**Properly paired reads (%)**
1	98.36	94.70
2	98.16	93.99
3	98.49	94.66
4	98.31	94.82
5	98.40	95.15
6	98.32	94.42

Article title: An effect of large-scale deletions and duplications on transcript expression. Journal name: Functional & Integrative Genomics. Authors: Magda Mielczarek[1], Magdalena Frąszczak[1], Anna E. Zielak-Steciwko[1], Błażej Nowak[1], Bartłomiej Hofman[1], Jagoda Pierścińska[1], Wojciech Kruszyński[1], Joanna Szyda[1].Affiliations: [1]Biostatistics group, Department of Genetics, Wroclaw University of Environmental and Life Sciences, Wroclaw, Poland. magda.mielczarek@upwr.edu.pl

Supplementary material S3. The average sequence quality of (a) raw and (b) filtered DNA samples.

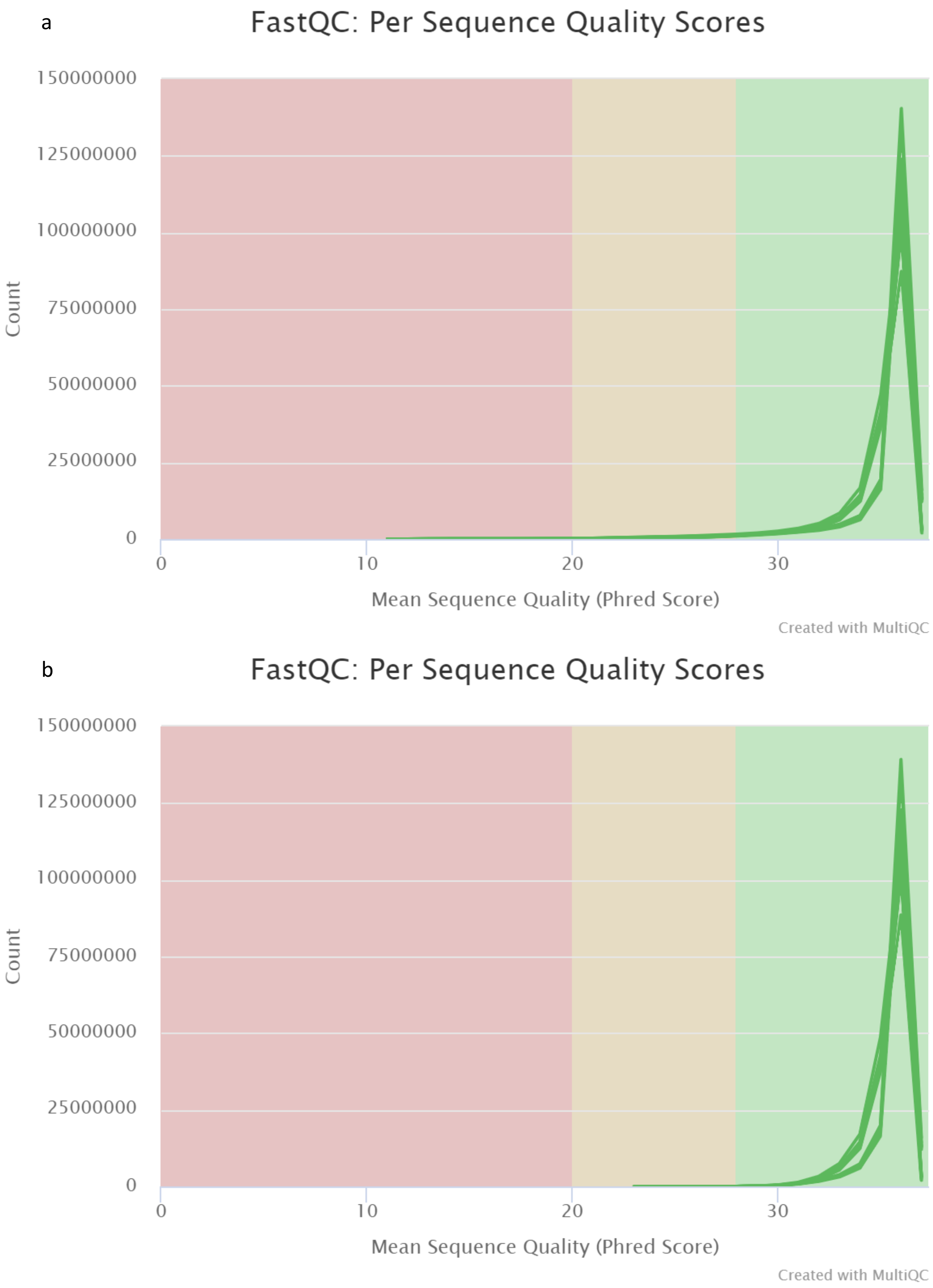

Article title: An effect of large-scale deletions and duplications on transcript expression. Journal name: Functional & Integrative Genomics. Authors: Magda Mielczarek[1], Magdalena Frąszczak[1], Anna E. Zielak-Steciwko[1], Błażej Nowak[1], Bartłomiej Hofman[1], Jagoda Pierścińska[1], Wojciech Kruszyński[1], Joanna Szyda[1].Affiliations: [1]Biostatistics group, Department of Genetics, Wroclaw University of Environmental and Life Sciences, Wroclaw, Poland. magda.mielczarek@upwr.edu.pl

Supplementary material S4. The average sequence quality of (a) raw and (b) filtered RNA samples.

a

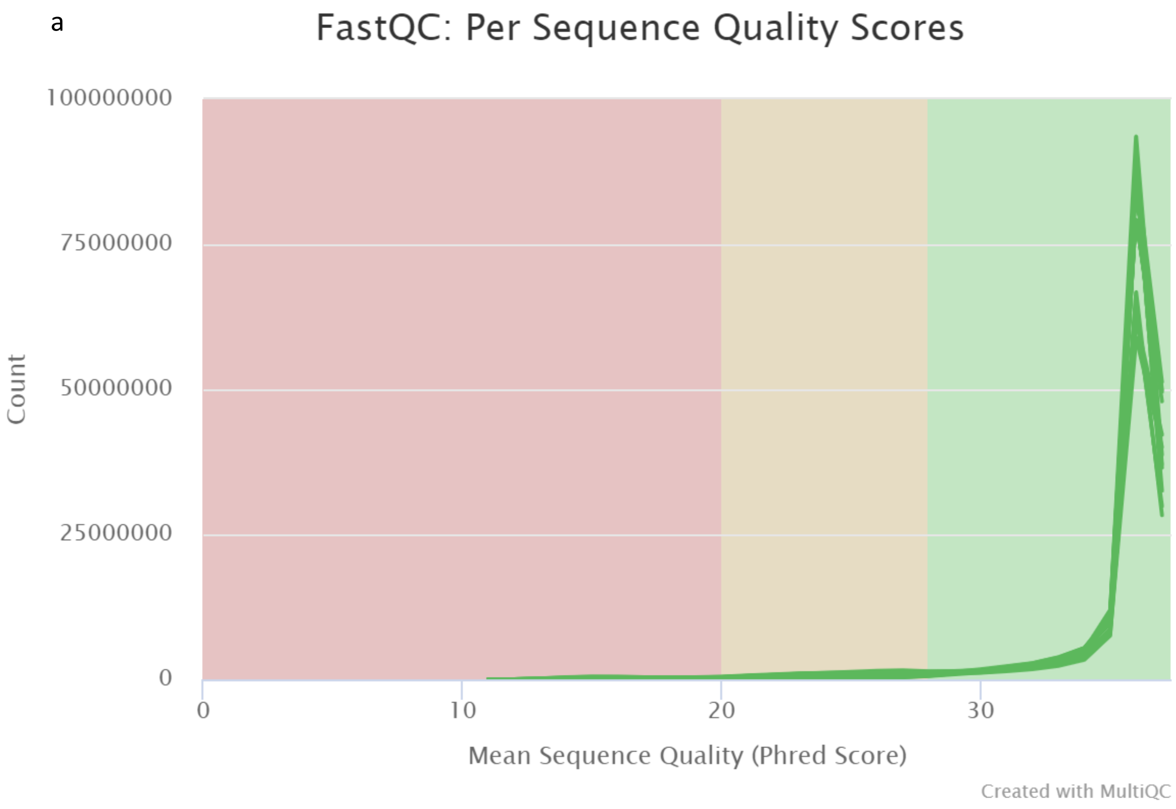

b

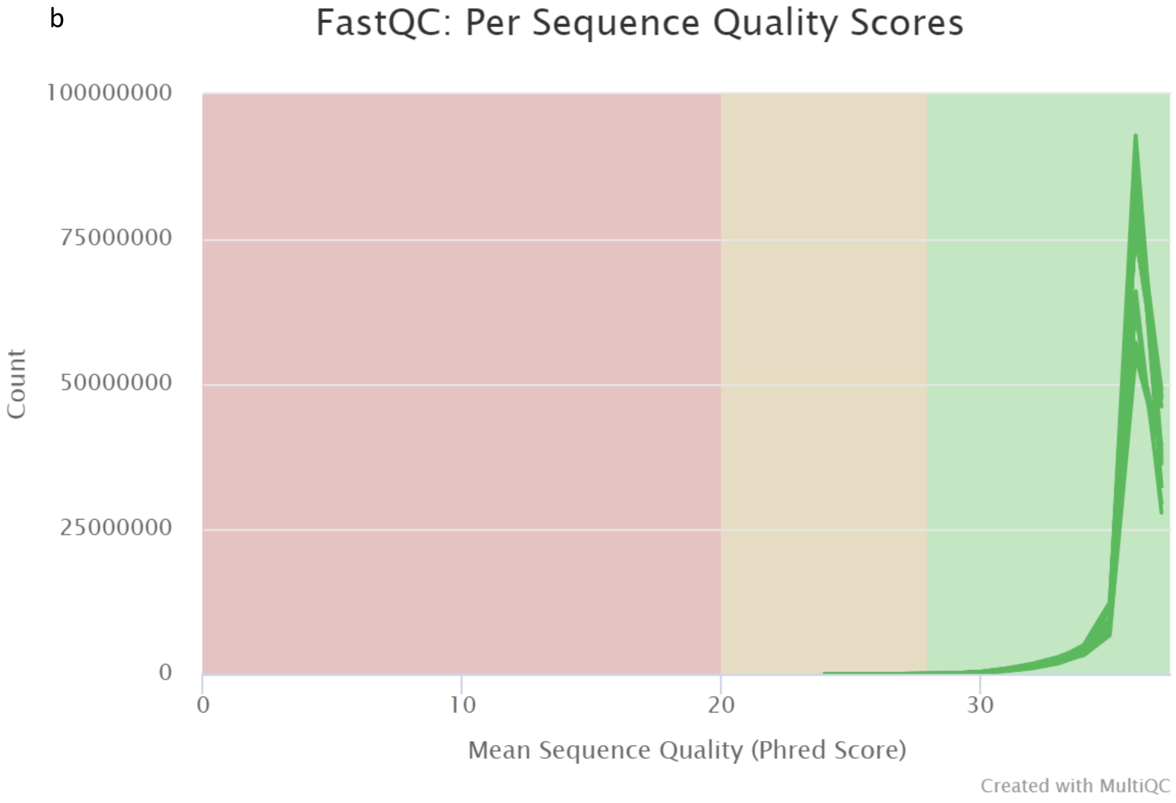

Supplement: Supplementary file 1 — Supplementary file1 (PDF 278 KB) [file 10142_2022_946_MOESM1_ESM.pdf]
